# Updates on Clinical Trials and Molecular Characteristics of Locally Advanced and Oligometastatic Renal Cell Carcinoma

**DOI:** 10.3390/ijms27052189

**Published:** 2026-02-26

**Authors:** Temitope M. Ogunmola, Zeng Jin, Jeremy Kleberg, Tanzia Islam Tithi, Seyedehalaleh Anvar, Chandra K. Maharjan, Jonathan Alexander Chatzkel, Sara Moscovita Falzarano, Ryan Kolb, Myung-Chul Kim, Weizhou Zhang

**Affiliations:** 1 Department of Pathology, Immunology and Laboratory Medicine, University of Florida College of Medicine, Gainesville, FL 32610, USA; temitopeogunmola@ufl.edu (T.M.O.); zengjin@ufl.edu (Z.J.); jkleberg@luc.edu (J.K.); ttithi@ufl.edu (T.I.T.); anvars@ufl.edu (S.A.); cmaharjan@ufl.edu (C.K.M.); sfalzarano@ufl.edu (S.M.F.); ryankolb@ufl.edu (R.K.); 2 UF Health Cancer Center, University of Florida, Gainesville, FL 32610, USA; jon.chatzkel@moffitt.org; 3 Department of Medicine Hematology and Oncology Division, University of Florida, Gainesville, FL 32610, USA; 4 Department of Urology, University of Florida College of Medicine, Gainesville, FL 32610, USA; 5 Laboratory of Clinical Pathology, College of Veterinary Medicine, Kyungpook National University, Daegu 41566, Republic of Korea

**Keywords:** oligometastatic disease, locally advanced cancer, stereotactic body radiation therapy, immune checkpoint inhibitors, molecular biomarkers, metastasectomy, tumor microenvironment

## Abstract

Renal cell carcinoma (RCC), particularly the clear cell subtype, is a solid tumor characterized by a high degree of immune cell infiltration, progressive immune dysfunction, and favorable response to immune checkpoint inhibitors (ICIs). Dual checkpoint inhibition or single-agent ICIs with anti-angiogenic therapies have become the standard of care in the treatment of advanced and metastatic RCCs. Locally advanced and oligometastatic RCCs are distinct clinical categories that are characterized by local invasion and initially limited metastatic spread, respectively, with different therapeutic responses and prognoses compared to either localized or advanced diseases. Herein, we summarize the biology, treatment, and molecular profiling of RCCs, providing an update on the clinical trials pertaining to cancer patients with locally advanced and oligometastatic RCC. We also discuss molecular features that could contribute to a better understanding of the tumor biology driving locally advanced and oligometastatic RCC.

## 1. Introduction

The term “renal cell carcinoma” (RCC) refers to malignant neoplasms originating from the renal epithelium. RCC accounts for more than 90% of all malignancies in the kidneys [1,2,3,4]. RCC ranks among the top ten causes of cancer-related deaths worldwide, according to GLOBOCAN estimates, making it a malignancy of significant concern [5]. In the United States, approximately 80,450 new cases of kidney cancer and 15,160 related deaths are projected in 2026, based on American Cancer Society statistics [6].

Several histologic subtypes of RCC are currently recognized, based on morphologic, immunophenotypical, and molecular features, with distinct biologic behavior and clinical implications [7]. Among them, the clear cell subtype (ccRCC) remains the most prevalent of these tumors, accounting for about 60–75% of RCC cases [8]. Advances in molecular investigations have helped identify the so-called “molecularly defined” RCC subtypes, including *TFE3*-rearranged RCC, *TFEB*-altered RCC, *ELOC* (formerly *TCEB1*)-mutated RCC, fumarate hydratase-deficient RCC, succinate dehydrogenase-deficient RCC, *ALK*-rearranged RCC, and SMARCB1-deficient renal medullary carcinoma [9,10].

**Figure 1:**
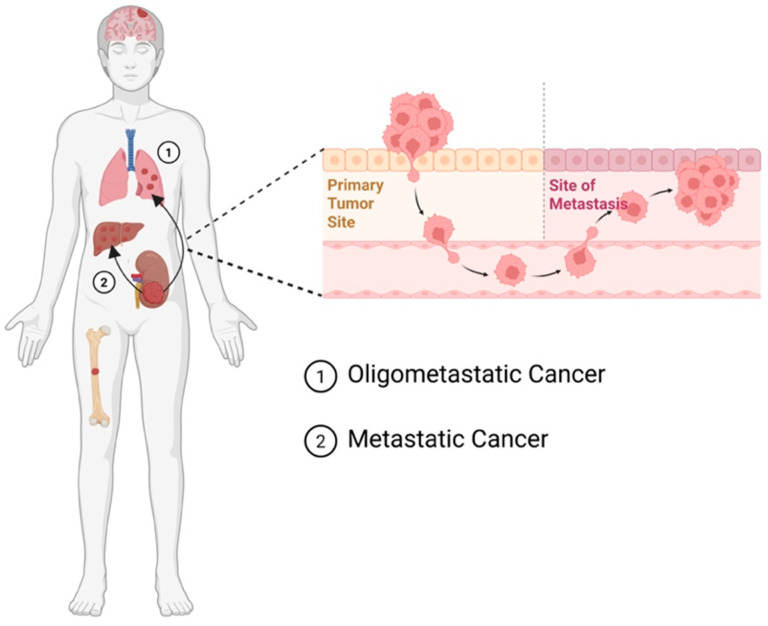
Oligometastasis of ccRCC. Oligometastasis refers to a clinical state in which a patient has a limited number of metastatic lesions, typically 1–5 sites, that are small in volume and often confined to one or a few organs. This state represents an intermediate stage between localized disease and widespread metastatic cancer. The biological features include but are not limited to less aggressive biology, fewer driver mutations or lower genetic complexity related to polymetastatic diseases, more robust immune surveillance, etc. Common sites include the lungs, liver, bone, lymph nodes, and adrenal glands.

RCCs exhibit varying degrees of cancer progression—localized and non-metastatic [11], high-risk or locally advanced, oligometastatic [12] or with widely disseminated metastases [13] (Figure 1). Locally advanced renal cell carcinomas (LARCCs) have been classified as cancers with either the spread of tumor cells to nearby tissues, such as the inferior vena cava and retroperitoneal lymph nodes [12,14,15,16,17,18], or those with local recurrence after radical surgery [12]. LARCC accounts for 4–10% of all RCC cases [12]. Due to LARCC’s tendency to invade surrounding tissues, it is categorized as either stage III or stage IV based on the extent of invasion according to the Tumor–Node–Metastasis (TNM) staging system (Table 1—TNM staging system of RCC) [19,20]. Approximately 20–30% of RCC patients present with synchronous metastatic disease [21], with common metastases to the lungs and lymph nodes, with other sites including bone, the liver, the adrenal glands and the brain [22]. Oligometastatic renal cell carcinomas (OMRCCs) refer to RCCs with limited (typically up to five) metastatic lesions or distal relapses [23]. OMRCCs represent a transitional phase between localized and widespread, metastatic renal cell carcinomas (mRCCs) [23,24]. As OMRCC involves metastasis, it is classified as a stage IV tumor according to the TNM staging system [25,26]. Given that 20–30% of patients present with metastasis at diagnosis [27], and that the majority of mRCC starts with single-site metastasis, this suggests that OMRCC precedes mRCC [28]. Moreover, OMRCC is a heterogeneous clinical state requiring individualized treatment strategies depending on oligometastatic disease characteristics [29,30].

Molecular features, such as the genomic and transcriptomic landscape for evaluating intrinsic cancer biology, aggressive biological behaviors, treatment response, diagnosis, and classification of RCC, have been reviewed [31,32,33,34,35]. Commonly identified genomic alterations involve *VHL*, *PBRM1*, *SETD2*, *KDM5C*, and *BAP1*, while other genes are less frequently altered, such as *TP53*, *ASXL1*, *PTEN*, *KDM5C*, *SPEN*, *NF1*, and *AKT1* [31,33,34,35,36].

The metastatic spread of RCC cells is thought to depend on the acquisition of a distinct phenotype by the primary tumor, accompanied by alterations in molecular and genetic profiles [37]. The establishment of oligometastatic lesions involves a multifaceted process encompassing the seeding and dissemination of cancer cells, selective homing to specific organs, interactions with the host microenvironment, and dynamic selection pressure [38]. Here, we will discuss current OMRCC and LARCC therapies and their molecular features that may inform patients’ therapy selection for improved therapeutic benefits. Specifically, we first outline the clinical spectrum of locally advanced and oligometastatic RCC and summarize current treatment approaches. We then review the biological and molecular features underlying disease progression and metastatic dissemination. Finally, we discuss emerging therapeutic strategies, including stereotactic radiotherapy and immune checkpoint inhibition, and highlight ongoing clinical trials that may refine patient selection and treatment sequencing.

**Table 1:** TNM staging system for RCC.

Stage	T (Primary Tumor)	N (Regional Lymph Nodes)	M (Distant Metastasis)
I [39]	T1a—≤4 cm, limited to kidney	N0	M0
	T1b—>4–7 cm, limited to kidney		
II [25,39,40]	T2a—>7–10 cm, limited to kidney	N0	M0
	T2b—>10 cm, limited to kidney		
III [25,41]	T1–T2, limited to kidney	N1	M0
	T3a—extends into renal vein or perirenal/renal sinus fat, not beyond Gerota’s fascia		
	T3b—extends into vena cava below diaphragm		
	T3c—extends into vena cava above diaphragm or invades its wall		
IV [39,41]	T4—invades beyond Gerota’s fascia and/or ipsilateral adrenal gland	N0–N1	M0
	Any T	Any N	M0
		Any N	M1

## 2. Crosstalk Between Renal Tumor and Its Microenvironment

Renal tumor initiation and progression are driven by both intrinsic tumor-cell alterations and extrinsic factors within the tumor microenvironment (TME) [42]. In the TME, bidirectional crosstalk between renal tumor cells and a diverse array of cellular and acellular components, including immune cells, cancer-associated fibroblasts, vascular endothelial cells, the extracellular matrix, and soluble mediators, collectively shapes tumor behavior, immune evasion, and metastatic potential [43].

Given that obesity is an established major risk factor for RCC [44], the contribution of adipose tissue to the perinephric microenvironment has become a growing area of investigation [45]. Perinephric adipose tissue, which is anatomically proximate to the kidneys, is particularly implicated in the progression of locally advanced disease. Wei et al. demonstrated bidirectional communication between ccRCC cells and perinephric adipose tissue that drives tumor growth and metastasis [46]. Specifically, ccRCC cells secrete parathyroid hormone-related protein, which induces the browning of perinephric adipose tissue, particularly beige adipocytes, resulting in elevated lactate production. This lactate is subsequently taken up by tumor cells and utilized to fuel tumor growth, invasion, and metastatic spread [46,47].

The molecular mediators of adipose tissue browning in this context include the transcriptional regulators UCP1, PRDM16, and PGCα, which govern the thermogenic and metabolic reprogramming of white adipose tissue into a beige phenotype [48]. This metabolic reprogramming of the tumor-adjacent adipose niche not only provides energetic substrates to cancer cells but also contributes to the immunosuppressive milieu that facilitates immune evasion and disease progression [43]. Together, these findings underscore the importance of considering the perinephric microenvironment as a therapeutic target and a contributor to the distinct biological behavior of locally advanced RCC.

## 3. Treatment Approaches for Oligometastatic RCC

Patients with oligometastatic disease can benefit from multidisciplinary management [49]. Given the complexity and biological heterogeneity of oligometastatic RCC, treatment strategies must balance systemic disease control with the potential benefits of localized interventions. While systemic therapy remains a primary treatment approach for mRCC, in select cases of OMRCC, clinicians may consider local therapies to potentially delay or defer systemic therapy, particularly for patients with favorable risk features, asymptomatic disease, and good performance status [23,50]. As a result, much consideration is given to approaches that minimize toxicities while maximizing the chance of a durable response [51].

In the metastatic setting, systemic therapy has evolved substantially over the past decade. Immune checkpoint inhibitor (ICI)-based combination regimens have demonstrated significant improvements in overall and progression-free survival in large phase III trials and are currently established as first-line systemic therapy for metastatic RCC [39]. These include dual ICI regimens (nivolumab plus ipilimumab; CheckMate 214), ICI/VEGFR-TKI combinations (axitinib plus pembrolizumab, KEYNOTE-426; cabozantinib plus nivolumab, CheckMate 9ER; lenvatinib plus pembrolizumab, CLEAR), and VEGFR-TKI/PD-L1 combinations (axitinib plus avelumab, JAVELIN Renal 101) [40,41,52,53]. However, in patients with oligometastatic disease, there is growing interest in more definitive treatment approaches, including surgical resection (metastasectomy), or ablative therapies, such as radiofrequency ablation or stereotactic body radiation therapy (SBRT), to target metastatic lesions [54]. Broadly, these treatments are categorized as metastasis-directed treatments (MDTs). These localized treatments can offer the potential for long-term disease control and even cure in select patients with oligometastatic RCC [55]. In recent times, the management of OMRCC has shifted to multimodal approaches where systemic therapies are combined with local approaches, addressing the limitations of single-modality approaches. Here, we will discuss certain treatment strategies for oligometastatic RCC.

## 4. Metastasectomy

Among metastasis-directed treatment strategies, surgical approaches remain an important consideration. Locoregional therapies play a critical role in the management of OMRCC, defined by limited metastatic sites (typically ≤3–5 lesions) [23], aiming to achieve local control and potentially prolong survival [55]. One such approach is metastasectomy, a locally directed strategy involving surgical resection of tumors at discrete metastatic sites with the goal of achieving a disease-free state. Metastasectomy plays a crucial role in the management of OMRCC. This approach is particularly considered in patients with favorable-risk features, limited metastatic burden, and good performance status, where complete resection of metastases may delay or eliminate the need for systemic therapy [56].

Cytoreductive nephrectomy (CN) serves a distinct role from metastasectomy in the management of metastatic RCC [57]. While metastasectomy targets metastatic lesions with the goal of achieving macroscopic clearance in oligometastatic disease, CN focuses on debulking the primary renal tumor to rescue overall tumor burden, alleviate symptoms, reduce tumor-driven immunosuppression, or potentially improve response to subsequent systemic therapies, particularly in the context of ICIs or targeted agents [57,58,59]. These two surgical approaches serve different clinical indications and should not be viewed as directly comparable interventions.

Regarding CN, the CARMENA trial, a phase III randomized trial, demonstrated that sunitinib monotherapy was noninferior to upfront CN followed by sunitinib in intermediate or poor-risk patients, which limits the applicability of its findings to the oligometastatic setting, where patients often have more favorable risk [60]. The ongoing PROBE trial is re-evaluating the role of upfront CN in the era of ICI combination therapy [61], and the NORDIC-SUN trial is comparing deferred CN following nivolumab plus ipilimumab or TK/ICC versus no CN [62].

Common metastatic sites amenable to resection include the lungs, pancreas, adrenal glands, liver and lymph nodes, which are often accessible in patients with limited disease and favorable-risk profiles [22,28]. In contrast, sites such as the brain and bone are less suitable for surgical intervention due to anatomical complexity and higher procedural risks, often necessitating alternative treatments like stereotactic body radiation therapy (SBRT) [63].

Evidence from retrospective and meta-analytic studies supports the role of metastasectomy in oligometastatic RCC. A meta-analysis conducted by Zaid et al. [64] found that complete metastasectomy, defined as resected tumor at all metastatic sites with no residual tumor, conferred a substantial survival advantage over incomplete resection or no surgical resection, with median overall survival improving from 36.5 to 142 months after complete resection versus 8.4 to 27 months with incomplete resection. Zaid’s review concluded that complete metastasectomy is more beneficial, reducing risks associated mortality than incomplete metastasectomy [64]. A separate meta-analysis demonstrated significantly improved overall survival in patients undergoing metastasectomy compared with non-surgical management (HR of 2.15, 95% CI = 1.59–2.92, I2 = 83%, *p* < 0.001) [65]. Additionally, a large cohort study showed that metastasectomy was more efficacious than targeted therapy alone in managing mRCC [66]. However, the absence of prospective randomized studies limits the strength of these conclusions, necessitating prospective trials to confirm these findings.

Metastasectomy has also been demonstrated to delay the initiation of systemic therapy [23]. With the advent of ICI-based regimens, there is growing interest in integrating MDT with systemic immunotherapy in OMRCC management, reshaping the treatment landscape [23]. The SURTIME trial which evaluated the sequencing of systemic therapy and CN in mRCC is discussed in greater detail in the VEGF-TKI section below.

## 5. Stereotactic Body Radiation Therapy (SBRT)

In addition to surgical resection, non-invasive ablative approaches have gained increasing attention in the oligometastatic setting. SBRT represents a paradigm shift in the management of OMRCC, offering the benefits of minimized toxicity and improved patient quality of life through the delay of systemic therapy [67,68]. SBRT is applicable not only to anatomically inaccessible lesions such as those in the brain and bone, but also to accessible sites including the liver, lungs, and lymph nodes, where it has demonstrated high local control rates with acceptable toxicity [69]. In contrast to metastasectomy, SBRT is a non-invasive ablative procedure that delivers fractionated high doses of radiation to metastatic sites, utilizing advanced imaging devices for precise delivery [70]. Beyond its established role in oligometastatic disease, SBRT continues to gain traction as a primary local treatment for patients with localized ccRCC who are medically inoperable due to comorbidities or advanced age, as well as those with small renal tumors, thereby broadening its clinical applicability [71,72].

Early work demonstrating that SBRT directed toward the lungs and liver achieved local control rates exceeding 90% with minimal toxicity [73] provided clinical support for its use in RCC, helping to overcome the radioresistance from the use of conventional fractionation [74]. Taken together, SBRT offers durable local control and functional response, rather than merely palliation across a spectrum of disease settings.

Dason et al. proposed that SBRT may be particularly beneficial for patients with a true oligometastatic state, characterized by indolent disease, who might otherwise be classified by IMDC criteria into favorable- or intermediate-risk groups warranting systemic therapy [23]. In an observational study, Zhang et al. found that SBRT effectively controlled oligometastatic lesions and significantly deferred the need for systemic therapy, contributing to improved patient quality of life [50]. Wersall et al. evaluated the SBRT outcomes in 58 patients with extracranial RCC, 50 with more than three metastases and 8 with inoperable primary tumors, and reported high local control rates with low toxicity [75]. Taken together, SBRT is promising and applicable where surgical interventions are challenging.

A phase II trial enrolled 30 patients with up to five metastatic lesions and no more than one prior systemic treatment [51]. All patients had undergone prior nephrectomy and were classified as intermediate or favorable risk by IMDC criteria. SBRT was delivered as up to five fractions with at least 7 Gy per fraction or treated with intensity-modulated radiation therapy at a dose of 60–70 Gy in ten fractions or 52.5–67.5 Gy in 15 fractions for lesions in locations deemed unsafe for ablative dosing [51]. The intent-to-treat endpoint and progression-free survival were achieved with limited adverse events. However, it should be noted that patients in this trial were not systemic-naïve, having previously received systemic therapy, which may confound the attribution of outcomes of SBRT alone. Prospective clinical trials exclusively enrolling systemic-treatment-naïve patients are therefore warranted. Such an approach was taken by Hannan et al., who assessed SBRT in 23 systemic-treatment-naïve patients with oligometastatic RCC in a prospective phase II study. With 75% of the patients in the intermediate-risk group, the study demonstrated that 91.3% of patients were free from systemic therapy at one year (95% CI: 69.5, 97.8), exceeding the pre-specified 60% > 1 year freedom from systemic therapy, and a 100% local control, one year progression-free survival, unaffected quality of life, no grade 3 or 4 toxicities, and overall survival of 95.7% (95% CI: 72.9, 99.4) [76]. These results provide compelling support for SBRT as a definitive, well-tolerated upfront strategy in select patients with oligometastatic RCC.

## 6. Biology of SBRT Efficacy in OMRCC Management

From a biological standpoint, SBRT synergizes with the immune system to induce tumor regression and achieve improved outcomes through an abscopal effect, in addition to its direct tumor-cytotoxic activity [77]. Conventional fractionated radiation therapy has historically been associated with limited efficacy in RCC, largely due to the intrinsic radioresistance [78,79]. The biological mechanism underlying this resistance is partly attributed to the p53-mediated apoptotic pathway, activated in response to DNA damage caused by reactive oxygen species (ROS) generated during repeated radiation fractions [80]. These fractions induce alternating cycles of hypoxia and reoxygenation, leading to bursts of ROS that damage cellular DNA. In most tumor types, this process sensitizes cells to apoptosis; however, RCC, particularly ccRCC, which is often characterized by von Hippel–Lindau (VHL) gene loss, responds differently. *VHL* loss results in the stabilization of hypoxia-inducible factors (HIFs), which in turn upregulate vascular endothelial growth factor (VEGF) and other pro-survival pathways. The resulting hypoxic tumor microenvironment and VEGF-driven angiogenesis support endothelial survival and inhibit apoptotic signaling, allowing tumor cells to evade cell death despite radiation-induced DNA damage [81]. This adaptation ultimately contributes to the observed radioresistance in conventional radiation therapy for RCC [77].

**Figure 2:**
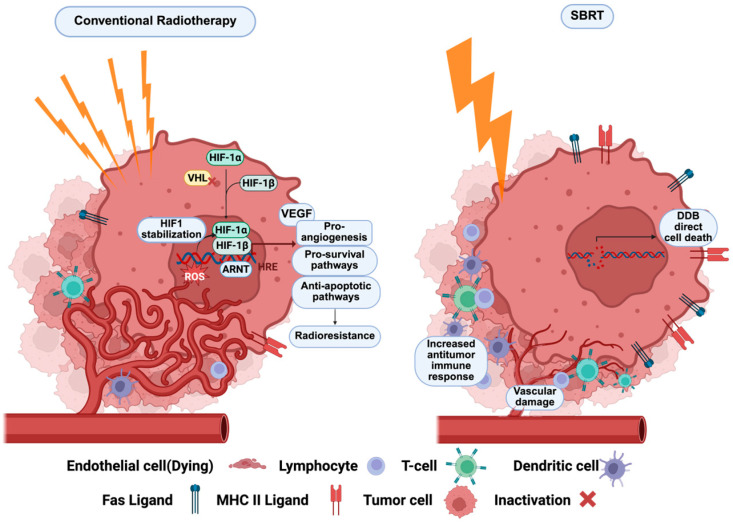
The biology of SBRT. SBRT delivers higher precision and accuracy, with shorter treatment duration and higher radiation doses relative to conventional radiotherapy, which provides superior local control, better sparing of normal tissues, limits neoangiogenesis, and has potential in stimulating cancer immunosurveillance.

SBRT on the other hand represents a biologically distinct approach to radiation delivery (Figure 2), capable of overcoming many of the limitations observed with conventional fractionated therapy in RCC. Unlike conventional therapy, which delivers small doses over many sessions, SBRT administers ablative doses of radiation in fewer, highly focused fractions [82], resulting in direct tumor cell killing through extensive DNA double-strand breaks that overwhelm the cell’s repair capacity, independent of p53-mediated apoptosis. Importantly, SBRT also disrupts the tumor vasculature, causing endothelial apoptosis and vascular collapse to reduce oxygen and nutrient supply, hence inducing ischemic tumor death [83]. In those RCCs, where VHL loss and VEGF-driven angiogenesis contribute to radioresistance, SBRT’s vascular-targeting effect is particularly advantageous. Moreover, SBRT elicits immunomodulatory effects, including the release of tumor antigens and pro-inflammatory cytokines that prime anti-tumor immune responses [84]. This immune activation may contribute to the “abscopal effect,” where tumors outside the radiation field also regress. Together, these biological mechanisms make SBRT a promising strategy in the management of oligometastatic RCC, especially in cases resistant to conventional radiation approaches [75].

Chow et al. [85] performed a high-throughput transcriptomic analysis of RCC samples from patients treated with SBRT, and confirmed that SBRT elicits a shift in the immune landscape. Their findings revealed that T cell repertoires expanded two weeks post-radiotherapy, coupled with elevated levels of the IL-2 family cytokines, highlighting the influence of single-dose radiation in facilitating an immune-sensitizing tumor microenvironment [85]. It is important to note, however, that the synergy between SBRT and immune checkpoint inhibitors in OMRCC remains a hypothesis supported by mechanistic and early clinical data rather than definitive demonstrated efficacy. The clinical combination is under active investigation (discussed below), and conclusions regarding synergistic benefits should be regarded as preliminary until confirmed by prospective randomized trials.

## 7. ICIs for the Treatment of OMRCC

Beyond local and ablative therapies, systemic immunotherapy plays an increasingly central role in the management of oligometastatic disease. In recent years, the introduction of ICIs has significantly improved therapeutic outcomes in mRCC, establishing ICI therapy as a standard of care [86]. Predictive biomarkers for ICI response in ccRCC remain an active area of research. Single-cell analysis of ccRCC has revealed a shift from M2-like to M1-like tumor-associated macrophages (TAMs) alongside CD8^+^ T cells exhibiting cytotoxic or progenitor-like phenotypes in ICI responders, contrasting with anti-inflammatory M2-like TAMs and exhausted CD8+ T cells in non-responders [87]. Immune regulatory genes including APOE, C1Q, and TREM-2 expressed by TAMs have been associated with ICI resistance [88]. Furthermore, Borcherding et al. identified a distinct population of CD39+ CD8+ T cells with an exhausted phenotype in ccRCC, whose presence correlated with higher histological tumor grades and poor overall survival, reflecting an immune-evasive TME [89]. In the context of oligometastatic RCC, the KEYNOTE-564 trial (phase III, double-blind) enrolled 994 patients with high-risk RCC, including a subgroup with M1 no-evidence-of-disease (M1 NED) status following complete resection of all metastatic lesions. All patients had undergone nephrectomy or complete metastasectomy within the prior 12 weeks. Adjuvant pembrolizumab significantly improved disease-free survival compared with a placebo, with the greatest relative benefit observed in the M1 NED subgroup. At 48 months, overall survival was 91.7% with pembrolizumab versus 86.0% with the placebo [90,91], supporting ICI therapy following complete surgical resection in a select oligometastatic population.

The RAPPORT trial, a small single-arm, multi-institutional phase II trial evaluated the efficacy of pembrolizumab (200 mg every 3 weeks for approximately 24 weeks) following SABR (20 Gy in a single fraction or 30 Gy in 10 fractions) to all metastatic lesions in 30 treatment-naïve patients with oligometastatic disease [92]. A high local control rate was achieved with an objective response rate of 38%, including durable complete responses. Notably, no patient required additional systemic therapy within the first year, highlighting the clinical relevance of targeting limited disease burden early. Mechanistically, the study suggested that SABR may prime the immune system by enhancing intratumoral CD8^+^ T cell infiltration, thereby creating an immunogenic microenvironment receptive to PD-1 blockade [92]. These findings are consistent with the hypothesis that locoregional and systemic immunotherapies may cooperate to intercept metastatic progression at an early phase. However, given the small sample size and single-arm design of RAPPORT, these results should be interpreted with caution, and further prospective randomized studies are warranted to establish whether this combination approach confers synergistic benefit.

## 8. VEGFR-TKI for the Treatment of OMRCC

The SURTIME trial evaluated whether deferred CN following upfront sunitinib yielded superior outcomes compared with upfront CN followed by systemic therapy. The study demonstrated that upfront sunitinib exposure followed by deferred CN improved overall survival compared with immediate CN before systemic therapy [93]. This trial is most applicable to the mRCC setting; however, its findings may inform the concept of sequencing systemic and surgical interventions in oligometastatic disease as well.

In oligometastatic settings, multimodal approaches combining VEGFR-TKI use with focal therapy have been explored to achieve disease control when disease progression occurs after SBRT monotherapy [94]. Yang Liu et al. 2021 evaluated the survival benefits of combining SBRT with VEGFR-TKI, which yielded improved overall survival compared to VEGFR-TKI alone in patients with oligometastasis [95]. This supports the rationale for evaluating VEGFR-TKI as adjuvant therapy following upfront SBRT in prospective settings, given that ablative therapy is increasingly considered in OMRCC management.

The ASCO guidelines recommend that patients who progress on combination immunotherapy such as nivolumab and ipilimumab should be offered subsequent therapy with a VEGFR-TKI in advanced RCC settings [96]. Whether this therapeutic sequence is directly applicable to the oligometastatic setting remains unclear and merits further investigation, given the distinct clinical trajectory of oligometastatic disease.

## 9. Treatment Approaches for LARCC

Treatments for LARCC have undergone significant advancements. This type of cancer lacks metastatic spread (Mo); however, it may spread into the tissue around the kidney (fascia), blood vessels such as the renal vein or vena cava, and nearby lymph nodes, or remain confined within the kidney. The main goal of the treatment for organ-confined LARCC is to check the progression targeting local mass.

Radical and Partial Nephrectomy (pre-2000s): The initial treatment option for locally advanced RCC was radical nephrectomy. This approach involves the removal of the entire kidney along with some surrounding tissues aiming to achieve local control of the tumor [12]. However, over the years, nephron-sparing surgery has become more popular for increasingly complex renal masses. This method sometimes shows clinical dilemma for patients with bilateral synchronous masses which require radical nephrectomies due to the size, complexity, and location of the tumor [97].

Introduction of Adjuvant Therapy (2000s): Targeted therapies, including sunitinib and sorafenib, were explored as adjuvant options following nephrectomy to reduce recurrence risk [97,98,99]. The ASSURE trial failed to demonstrate a significant improvement in disease-free survival (DFS) with adjuvant sunitinib or sorafenib [100,101]. In contrast, the S-TRAC trial demonstrated that adjuvant sunitinib improved DFS in high-risk post-nephrectomy patients [102], supporting regulatory approval of adjuvant sunitinib in this setting; however, overall survival benefit was not demonstrated, and toxicity concerns have limited its widespread adoption. The contrasting results of these trials may reflect differences in patient selection criteria and disease heterogeneity.

Neoadjuvant Therapy (2010s): Neoadjuvant therapy comprising VEGF-targeted agents prior to surgery for locally advanced RCC was explored to downstage unresectable tumors before surgery to achieve better surgical outcomes. VEGF inhibitors, such as sunitinib and pazopanib, have been investigated as strategies to facilitate tumor downstaging prior to surgery; this approach remains investigational and has not demonstrated a survival benefit in prospective randomized trials [103]. Prospective trials such as PROSPER have not demonstrated improvement in recurrence-free survival with perioperative ICI strategies. In patients with unresectable, locally advanced disease, systemic therapy is employed for disease control with palliative intent, with the goal of achieving durable response to enable consolidative surgery in selected cases. One of the major concerns of preoperative therapy with targeted therapy for locally advanced RCC is wound complications. Agents such as bevacizumab (VEGF antibodies) have been identified to show significant wound complications and increased morbidity [104]. 

Introduction of ICIs (2020s): ICI treatment has been explored in patients with locally advanced RCC to control the recurrence after surgical resection [105]. Recently, the KEYNOTE-564 trial has demonstrated that pembrolizumab (anti-PD-1 monoclonal antibody), administered as adjuvant therapy after nephrectomy, can lead to clinically significant improvement in overall survival in patients with increased risk of recurrence compared to a placebo [106].

Combination Therapy—ICIs and VEGF inhibitors: Combination of ICIs with VEGF inhibitors has been explored to improve surgical resectability and long-term outcomes [107]. Promising efficacy has been demonstrated by the combination therapy of axitinib and ICIs [108]. Trials are also evaluating triplet therapies, which include ICIs, TKIs, and other novel agents, such as pembrolizumab, lenvatinib, and belzutifan (NCT04976634), which may eventually result in more alternatives in the first-line treatment plan [109].

## 10. Clinical Trials for LARCC and OMRCC (Table 2)

For LARCC, surgery is the primary treatment option. However, despite nephrectomy, patients remain at risk for metastatic disease. Numerous clinical trials have been performed to evaluate the potential benefits of immunotherapy post-nephrectomy to improve survival outcomes. 

The IMmotion010 trial (NCT03024996) explored the effectiveness of atezolizumab (anti-PD-L1) as adjuvant therapy in patients at high risk of recurrence following kidney removal. Patients with atezolizumab treatment after nephrectomy demonstrated no benefits in disease-free survival compared to surgery alone [110]. The PROSPER trial (NCT03055013) evaluated localized and advanced RCC patient survival and recurrence-free benefits of nivolumab (anti-PD-1) administered before and after nephrectomy [111]. The study showed that there is no difference in recurrence-free survival between the nivolumab-treated group (either before or after nephrectomy) and surgery only with surveillance in high-risk patients. CheckMate 914 (NCT03138512) examined nivolumab, with or without ipilimumab, in patients with high-risk localized and advanced RCC postoperatively. Both part A (nivolumab + ipilimumab) and part B (nivolumab monotherapy) demonstrated no significant improvement in disease-free survival compared to a placebo [112]. However, the KEYNOTE-564 trial (NCT03142334), a phase III study that examined pembrolizumab (anti-PD-1) as an adjuvant treatment for high-risk RCC after surgery, showed that adjuvant pembrolizumab treatment results in significant improvement in overall survival compared to a placebo [113]. The RAMPART trial (NCT03288532), a phase III study, is investigating the use of durvalumab (anti-PD-1) alone or in combination with tremelimumab (anti-CTLA-4) as post-nephrectomy adjuvant therapies. In this trial, 1750 patients with all cell types of RCC who underwent resection of the tumor were randomized into three groups: active surveillance, anti-PD-1 therapy, or a combination of anti-PD-1 and anti-CTLA-4. The results of the trial have not yet been reported [114].

Beyond immunotherapy, targeted therapies are also being evaluated for survival benefits. The ATLAS trial (NCT01599754) evaluates axitinib (a TKI) as an adjuvant treatment in high-risk localized and advanced disease after surgical kidney removal. Even though the results showed no overall disease-free survival, there is an improvement in the highest-risk subpopulation [115]. The EVEREST trial (NCT01120249) studied the mTOR inhibitor everolimus as adjuvant therapy, focusing on recurrence-free survival. The results showed postoperative everolimus treatment did not improve patient outcomes [116]. The ASSURE trial (NCT00326898) also assessed the efficacy of adjuvant-targeted therapies such as sunitinib and sorafenib in high-risk RCC patients. Both treatment groups did not demonstrate any improvement in disease-free survival or overall survival [117].

Finally, NeoAvAx (NCT03341845), a phase II trial, recruited patients with locally advanced kidney cancer to assess the effect of the combination of avelumab with axitinib on tumor size and patient outcomes.

For OMRCC, several clinical trials are exploring potential treatment strategies for patients with a limited number of metastases. The phase III SABLOR trial (NCT06467097) is evaluating whether adding radiotherapy to standard systemic immunotherapy can improve outcomes. In this trial, patients with fewer than five detectable metastases are randomized to receive either standard immunotherapy alone or in combination with SBRT. Key endpoints include 1-year progression-free survival, adverse events, and overall survival rates.

Another study, SAbR (NCT02933242), is a study with radiation therapy investigating the use of stereotactic ablative body radiation (SAbR) to treat up to three metastases in patients with oligometastatic RCC. This trial aims to assess progression-free survival and overall survival, focusing on how local radiation therapy can manage metastatic sites and delay disease progression.

**Table 2:** Clinical trials for LARCC and OMRCC.

Study Name	Identifier	Subtype	Agent	Target	Control	Total	Citation
RAMPART	NCT03288532	LARCC	Durvalumab, tremelimumab	Anti-PD-1, anti-CTLA-4	Active surveillance	1750	[114]
KEYNOTE-564	NCT03142334	LARCC	Pembrolizumab	Anti-PD-1	Placebo	994	[113]
IMmotion010	NCT03024996	LARCC	Atezolizumab	Anti-PD-L1	Placebo	778	[110]
PROPSER	NCT03055013	LARCC	Nivolumab	Anti-PD-1	Active surveillance	819	[112]
CheckMate 914	NCT03138512	LARCC	Nivolumab, ipilimumab	Anti-PD-1, anti-CTLA-4	Placebo	1641	[116]
ATLAS	NCT01599754	LARCC	Masatinib	TKI	Placebo	724	[115]
EVEREST	NCT01120249	LARCC	Everolimus	mTOR inhibitor	Placebo	1545	N/A
ASSURE	NCT00326898	LARCC	Sorafenib, sunitinib	TKI	Placebo	1943	[101]
NeoAvAx	NCT03341845	LARCC	Avelumab, axtinib	Anti-PD-L1, TKI	N/A	40	[118]
SABLOR	NCT06467097	OMRCC	Atereotactic ablative body radiation	Radiotherapy	Standard therapy	88	N/A
SAbR	NCT02933242	OMRCC	Stereotactic ablative body radiation	Radiotherapy	N/A	399	[119]

N/A = Not applicable.

## 11. Molecular Features of LARCC and OMRCC and Clinical Implications

Molecular features of locally advanced tumors, including gastric cancer [120], esophageal squamous cell carcinoma [121], colon cancer [122], and breast cancer [123], have been investigated and reported in recent years. Similarly, molecular profiling of oligometastatic disease has been studied in multiple tumor types [124]. Currently, emerging studies have reported molecular features typical of LARCC and OMRCC that could be driving these intermediate clinical spread states of RCC. Molecular features reported across studies with clinical relevance are presented below.

Braun et al. [125] characterized an immune-engaged but functionally constrained microenvironment in LARCC using single-cell RNA sequencing (scRNA-seq) of tumor-infiltrating leukocytes across advancing ccRCC stages, including four patients with locally advanced (stage III) disease. The study found that terminally exhausted CD8^+^ T cells and suppressive M2-like tumor-associated macrophages (TAMs) were increased during cancer progression, whereas proinflammatory M1-like macrophages were decreased. Of note, the study revealed that the interactome signature derived from exhausted CD8^+^ T cells and M2-like TAMs could represent progressive immune dysfunction with advancing disease stage in ccRCC, including LARCC. The molecular interaction signature consisted of 126 genes, including *PDCD1*, *CTLA4*, *CXCL16*, *CXCR4*, *CD74*, *MIF*, *VCAM1*, a4b1 complex, *LAGALS9*, *CXCL12*, and *CSF1R*, among others, which taken together inversely correlate with worse prognosis. Collectively, these molecular features indicate that LARCC is immune-engaged but functionally restrained, dominated by exhausted T cells and suppressive myeloid programs, which may explain why selected patients can still respond to immune checkpoint blockade despite limited effective systemic immune escape.

In LARCC with inferior vena cava (IVC) thrombus, Annels et al. [126] linked immune exhaustion to metabolic impairment during progression. Consistent with previous findings that RCC is manifested by T cell exhaustion [88,125,127,128], the proportion of tumor-infiltrating CD4^+^ and CD8^+^ T cells with PD1 expression was significantly increased during the local progression from peripheral blood and pT1/2 to locally advanced disease. Of note, some ccRCC patients with IVC thrombi were found to have a high proportion of tumor-infiltrating CD8^+^ T cells with the expression of inhibitory receptors TIM3 and LAG3, which tended to increase in more advanced metastatic stages. Importantly, this exhausted immune context was accompanied by metabolic dysfunction, including depleted mitochondrial mass and coordinated downregulation of metabolic genes during progression into IVC thrombus (e.g., *NOX4*, *HIF1A*, *PEGFRB*, *RAC2*, *G6PD*, *PDFGRA*, *SLC38A2*, *FOXO3*, *HK1*, and *IDH1*). Some genes were significantly associated with clinical outcomes, e.g., *FOXO3* with a poor outcome and *G6PD/RAC2* with improved overall survival. Likewise, progression into metastatic disease compared to pT3-4 showed significant downregulation of several metabolic genes associated with a better or poor clinical outcomes, including *DLAT*, *ACO1*, *PLD1*, *PFKM*, *FASN*, *ALDOB*, *SLC2A2*, and *EIF4EBP1*, although the study did not specify whether the six ccRCC patients with distant metastases were classified as oligometastatic. This pattern suggests that LARCC can be locally aggressive while remaining metabolically constrained for efficient systemic dissemination, supporting aggressive local therapy in biologically selected patients.

Na et al. [20] implicated recurrence mechanism by selecting 312 cases of localized ccRCC from TCGA (The Cancer Genome Atlas) kidney clear cell renal carcinoma (KIRC) database. The study found that locally advanced (pT3-4) ccRCC cases with altered DNA-damage response (DDR) pathway were associated with decreased DFS compared to those without alteration in the DDR pathway (median, 123.7 vs. 23.0 months, *p* = 0.031). The association was more prominent in cases of pT3a LARCC. The DNA-damage-associated genes included *CHEK1*, *CHEK2*, *RAD51*, *BRAC1*, *BRACA2*, *MLH1*, *MSH2*, *ATM*, *ATR*, *MDC1*, *PARP1*, and *FANCF*, which might be associated with poor outcome due to increased recurrence in LARCC. DDR pathway alterations may promote genomic instability and subclonal diversification, suggesting local invasion and recurrence prior to acquisition of full metastatic competence and thereby supporting the concept of locally advanced RCC as an intermediate evolutionary state rather than a purely localized disease [129]. Accordingly, these molecular alterations may serve as prognostic biomarkers potentially identifying LARCC patients at higher risk of recurrence.

Some patients after ICB therapy have experienced a dramatic reduction in tumor thrombus size [130,131,132]. Shi et al. [128] investigated the single-cell tumor microenvironmental landscape of primary and locally advanced ccRCC with IVC thrombus. Overall, the study has shown the critical involvement of extracellular matrix (ECM) remodeling and angiogenesis in the generation and maintenance of vena cava thrombosis. IVC thrombi had more infiltration of tissue-resident CD8^+^ T cells with a high progenitor exhausted score, although primary ccRCC and the LARCC were both immune-suppressive, characterized by prominent infiltration of terminally exhausted CD8^+^ T cells. Of note, the study evaluated the prognostic value of molecular signatures derived from key players associated with immune modulatory and metastatic properties of LARCC. For example, using the external CheckMate 025 ccRCC cohort, the pre-therapy levels of IVC-resident CD8^+^ T cell signature were validated to predict better response to anti-PD-1 therapy through improved progression-free survival (PFS). Of note, high levels of *NDUFA*4*L*2 together with angiogenic and stromal programs, including *KDR* and *COL1A*1, were inversely associated with PFS and clinical outcome in the TCGA KIRC cohort, suggesting a hypoxia-adapted vascular niche linked to aggressive LARCC biology. Thus, NDUFA4L2-high endothelial programs may reflect a hypoxia-adapted vascular niche associated with poorer outcomes in LARCC.

López et al. [127] explained heterogeneous therapy response by focusing on intratumoral heterogeneity of ccRCC and performed spatial whole-transcriptome profiling of 19 intratumoral and peritumoral regions of two selected highly immunogenic stage pT3a (Grade 3) and stage pT3a (Grade 4) non-metastatic LARCCs. The study revealed a significant existence of intratumoral heterogeneity, supporting previous results that the tumor interior displays a more aggressive and metastasizing subclonal growth phenotype as well as an inverse correlation with therapeutic response [133,134,135]. Compared to the periphery, genes differentially expressed in the interior tumor (e.g., *SOD2*, *HLA*-*E*, *SERPINA3*, *BCL7B*, *CCL19*, *CCDC13*, *SPATA12*, *ST3GAL2*, *MEF2C*, *STEAP4*, and *KCNA4*) were significantly enriched in two pathways—one with run-related transcription factor 1 (RUNX1)-mediated regulation of megakaryocyte differentiation and the other with EIF2AK4 to amino acid deficiency. Of note, cell deconvolution analysis inferred preferential infiltration of Tregs and endothelial cells within the LARCC compared to the periphery, suggesting the involvement of immune suppression and angiogenesis in the RCC subtype. This spatial heterogeneity, arising from the underlying molecular features, may explain incomplete responses to systemic therapy, providing a rationale for multimodal treatment combining systemic therapy with definitive local control in LARCC.

Wuttig et al. explained metastatic transition by performing RNA-seq-based transcriptomic profiling of pulmonary metastatic ccRCC from 18 patients treated with nephrectomy. Based on clinicopathologic data, the study classified ccRCC patients into four subgroups: early metastases (disease-free interval (DFI) of a month, *n* = 6), late metastases (DFI of 92.5 months, *n* = 5), few metastases (≤8 metastatic lesions, *n* = 10), and multiple metastases (≥16 metastatic lesions, *n* = 7). The study found that early and late ccRCC could be distinguished by expression profiling of 306 genes (e.g., *HSPG2*, *ICAM2*, *PECAM1*, *SFTPC*, *TPM2*, and *PSPAN7*), which were mainly upregulated in the late-metastasis subgroup and significantly enriched with tumor and metastasis-associated gene sets, such as angiogenesis, cell migration, and adhesion. Interestingly, the ccRCC patients from the late-metastasis subgroup were all oligometastatic (≤7 metastatic lesions). The subgroups between few and multiple metastases were distinguished by 135 genes (e.g., *RASGEF1A*, *AGR3*, *CEACAM6*, and *GRP64*). Of note, the oligometastasis-associated molecular feature was significantly associated with varying growth potential but not angiogenesis during ontology analysis. Among the molecular features, an eleven-gene signature, including *AGR3*, *E2F8*, *EHF*, *GPR64*, *PBK*, *RASGEF1A*, *SCGB1A1*, *SCGB3A2*, *SFTPG*, *NKX2*-*1*, and *TMEM178*, had prognostic value to predict the number of metastases per patient [136]. Therefore, molecular stratification based on these features may help distinguish patients suitable for metastasis-directed therapy from those requiring systemic treatment escalation.

Molecular features have been utilized to study metastasis [32,137,138,139], evaluate prognosis [34,140], classify molecular subtypes [141], and predict clinical outcome to checkpoint and angiogenesis blockade in patients with RCC [142]. Although not specifically for LARCC and OMRCC, studies have reported biomarkers and gene signatures associated with advancing stage in RCC, which could be closely involved in the tumor biology driving those intermediate states.

Bhalla et al. [143] explained molecular staging by utilizing support-vector-machine-based supervised learning to identify gene-expression-based biomarkers that could discriminate early (pT1/2) and late (pT3/4) clinical status of ccRCCs collected from the TCGA KIRC cohort. The study provided a high classification performance of 28 genes to distinguish early- (e.g., *NR3C2*, *ENAM*, *C1orf69*, *BMP5*, *DNASE1L3*, *FRMPD2*, and *NBEA*) and late-stage RCC (e.g., *PLEKHA9*, *IRF7*, *TMEM214*, *PITX1*, *PLA2G2A*, *MAP6D1*, *SMPD4*, and *C11orf73*). These genes were significantly enriched in the cancer hallmark biological process of the PI3K-Akt signaling pathway. Meanwhile, *NR3C2* belonged to the molecular feature of oligometastasis, as provided by Wuttig et al. [136], suggesting growth potential of tumor cells during early and oligometastatic stages. Thus, these signatures may support molecular staging that reveals aggressive tumor biology beyond conventional pathologic classification. Similarly, Jones et al. [144] identified 31 genes with a continuous deregulation pattern as ccRCC progresses (normal kidney tissue versus early tumor stage (T1) and then T1 versus metastasis). Although the study focused on gene lists that could predict metastasis of RCC, we found that several genes overlapped in the gene lists associated with progressive immune dysfunction [125] (*CAV1* and *HLA*-*DQA1*), oligometastasis [136] (*KIAA0101*), IVC thrombus [128] (*ANXA4*, *LAPTM5*, and *AGTR1*), and intratumoral heterogeneity [123] (*MARCKS*), supporting the relevance of this gene set for LARCC. Similarly, Borys et al. [145] showed 36 genes, including *LOX*, associated with intratumoral heterogeneity of LARCC [127], that discriminate the two clinical stages, pT1 versus pT3 RCC, showing enrichment of molecular transporters, channel and transmembrane proteins. Thus, collectively, these transcriptomic overlaps indicate that molecular programs driving immune dysfunction, invasion, and heterogeneity emerge before overt metastasis, supporting the concept that LARCC represents a biologically progressive pre-metastatic state rather than a strictly localized disease.

Raghubar et al. [146] extended the immune-checkpoint trajectory across tumor progression using spatial transcriptomics of para-tumoral, low-grade, and high-grade ccRCC samples. This study found that tissue-resident monocytes (TIMs) surrounded the exhausted CD8^+^ T cells in high-grade LARCC. Of note, the TIMs showed a consistent increase in the expression of *HAVCR2* and *LAG3* checkpoint genes during low- to high-grade ccRCC progression. Meanwhile, there was no consistent increase in the expression of *PD*-*1*, *PD-L1* and *CTLA4* checkpoints and angiogenic genes during ccRCC progression. Lindner et al. [147] demonstrated prognostic value of *CXCR3* expression in association with advanced tumor stage, such as recurrence of localized RCC. Predominant expression of CXCR3 was identified in TILs and dendritic cells. Given that CXCR3 was listed amongst the molecular features of progressive immune dysfunction during advancing disease stage in ccRCC [125], CXCR3^+^ CD8^+^ T cells could be associated with the tumor biology of LARCC. Together, these immune features may function as predictive biomarkers for therapeutic response and disease progression in locally advanced RCC.

Taken together, LARCC and OMRCC represent an intermediate evolutionary state between localized and systemic disease, characterized by immune engagement with functional exhaustion, incomplete metabolic adaptation, and developing metastatic niche formation. Therefore, integrating molecular features into clinical decision-making may allow patient selection for surgery, immunotherapy, or combined multimodal treatment rather than relying solely on anatomical staging.

## 12. Conclusion and Future Directions

Patients with LARCC still have a significant risk of disease recurrence and progression, even after complete surgical resection and neoadjuvant therapy [12,148]. Oligometastatic state is associated with a relatively favorable outcome for RCC patients with bone metastasis after anti-angiogenic therapy [149]. Despite extensive investigations, no validated molecular predictive biomarkers of response to targeted therapy, immunotherapy, or to locoregional approaches have been rendered available in RCC [150,151,152,153], which could be attributed to high biological and clinical heterogeneity [13]. A greater understanding of the tumor biology driving LARCC and OMRCC is an unmet need. Accumulating evidence has shown that a certain stage of RCC progression appears to accompany a tipping point toward more aggressive phenotypes [127]. Molecular investigations summarized in this review indicate that LARCC and OMRCC are characterized by variable proliferative and angiogenic capacities of tumor cells with progressive immune dysfunction [125,126,127,128,136]. Future studies should consider the application of spatial transcriptomic analysis of tumor and immune heterogeneity with a focus on RCC cell subsets, their phenotypes, and functions, as well as the complex cell–cell interactions in the tumor microenvironment, separating the locally advanced and oligometastatic subtypes.

## Data Availability

No new data were created or analyzed in this study. Data sharing is not applicable to this article.
